# Research on cutting mechanism and process optimization method of gear skiving

**DOI:** 10.1038/s41598-025-88469-4

**Published:** 2025-02-03

**Authors:** Peng Wang, Yuanchao Ni, Xiaoqiang Wu, Jiaxue Ji, Geng Li, Jiahao Wu

**Affiliations:** 1 Tianjin High-end Intelligent Machine Tool Engineering Research Center, Tianjin University of Technology and Education, No 1310, Dagu South Road, Tianjin, 300222 People’s Republic of China; 2 College of Engineering, Inner Mongolia Minzu University, Tongliao, 028000 People’s Republic of China; 3 Postdoctoral Research Station in Mechanical Engineering, Tianjin University, Tianjin, 300182 People’s Republic of China

**Keywords:** Gear skiving, Cutting force, Cutting temperature, Process parameters, Genetic algorithm, Multi-objective optimization, Mechanical engineering, Software

## Abstract

The cutting force and cutting temperature have a significant impact on the service life and durability of gear skiving cutters. Due to unreasonable design, the existing process parameters lead to dramatically nonuniform cutting force and cutting temperature, which aggravates the rapid wear of gear skiving cutters. To address this issue, this paper first establishes a finite element model of skiving the internal circular arc tooth in pin wheel housing, and the simulation model is simplified to improve computation efficiency. Next, the impact of single process parameter on cutting force and cutting temperature is analyzed by controlling variable. Then, an orthogonal experiment is designed and the method of range analysis is employed to evaluate the significance of each process parameter. Furthermore, a prediction model of cutting force and cutting temperature is established using a neural network optimized by genetic algorithm. This prediction model allows for the construction of a multi-objective optimization model for the process parameters. By solving this model, the optimal combination of process parameters within the given ranges can be obtained to achieve reasonable and balanced cutting force and cutting temperature.

## Introduction

RV reducer is a widely used transmission device in the field of robot^1^. The pin wheel housing, as the crucial component of RV reducer, plays a significant role to ensure efficient power transmission^2^. Currently, the main machining process for the internal circular arc tooth of pin wheel housing is gear shaping. However, the cutting edge of gear shaping cutter has theoretical errors^3^, which affects the machining accuracy of the internal circular arc tooth. In contrast, the gear skiving cutter has no theoretical error, and can offer higher machining efficiency and around 2 to 3 times faster than gear shaping. Consequently, the gear skiving is a promising direction for machining the internal circular arc tooth in the pin wheel housing. Previous studies have demonstrated the feasibility of gear skiving for the internal circular arc tooth of the pin wheel housing in theory. However, the current skiving process suffers from unreasonable parameter design, resulting in excessive cutting force and cutting temperature for the skiving cutter. This issue not only diminishes the surface processing quality of the workpiece^4^, but also reduces the service life of skiving cutter. Unfortunately, the optimization model for the process parameters has not been established, which hinders the ability to provide theoretical guidance for reducing cutter wear. Under this background, it is crucial to investigate a parameter optimization method for skiving the internal circular arc tooth in pin wheel housing.

In recent years, several scholars have conducted extensive research on the cutting mechanism and design method of skiving cutter. J. Li et al.^5^ established a mathematical model for cutting force using micro-segment edges and proposed a calculation method for total cutting force based on the law of conservation of energy. H. Guo et al.^6^ examined the influences of skiving cutter parameters on the machined tooth surface of face gear, employing the kinematic principle of gear skiving to correct the tooth surface deviation. G. Zheng et al.^7^ analyzed the cutting force of skiving cutter under different feeding methods and proposed an improved multi-side feed technology, effectively reducing the wear of the cutting edge. E. K. Guo et al.^8^ proposed a design method for a new skiving cutter composed of both roughing blades and a finishing blade, analyzing the influence of the outside diameter on cutting force and the cross angle on chip formation and tool wear. This research greatly enhanced the productivity and the service life of skiving cutter. K. Fritz et al.^9^ studied chip thickness of gear skiving in a simulation model, further analyzed the relationship between skiving cutter wear and chip thickness, and optimized the process parameters of gear skiving to some extent. J. Christopher et al.^10^ developed a simulation model for gear skiving and compared the simulated and actual chip thickness, providing a basis for the subsequent research on the wear theory of skiving cutter. I. Masatomo et al.^11^ successfully predicted chip shape in gear skiving by calculating the volume of the workpiece and the sweeping volume of the cutting edge, achieving visualization of the machining process.

The above researches provide valuable references for this paper. However, the relationship between process parameters of skiving internal circular arc tooth and cutting force and cutting temperature remains unclear. To solve this problem, this article constructs a simplified finite element simulation model based on the kinematics principle of gear skiving. Through experimental data obtained from single-factor and orthogonal experiments, the influence of different process parameters on cutting force and temperature is analyzed. Then, a prediction model for cutting force and cutting temperature and an optimization model of process parameters are established, providing guidance for reducing cutter wear.

## Physical simulation modeling of gear skiving processing

In order to investigate the correlation between process parameters, cutting force and cutting temperature during the gear skiving process, a simulation model is developed using finite element technology.

### Gear skiving cutter design

Taking the pin wheel housing in RV40E reducer as a reference, the main parameters of the workpiece are shown in (Table 1). Accordingly, the parameters of the skiving cutter are proposed as shown in (Table 2).

**Table 1:** Parameters of internal circular arc tooth.

Teeth number	Arc radius/mm	Distribution diameter/mm	Central angle/°	Tooth width/mm
40	2.9	128	162.2	20.6

The skiving cutter used in the experiment is consisted of three main parts: the cutting edge, the rake face and the flank face. Among them, the cutting edge is formed by fitting the discrete points obtained by the intersection of the conjugate surface and a plane by the method of cubic B-spline^12^.

**Table 2:** Parameters of skiving cutter.

Teeth number	Rake angle/°	Relief angle/°
22	10	6.5

**Fig. 1:**
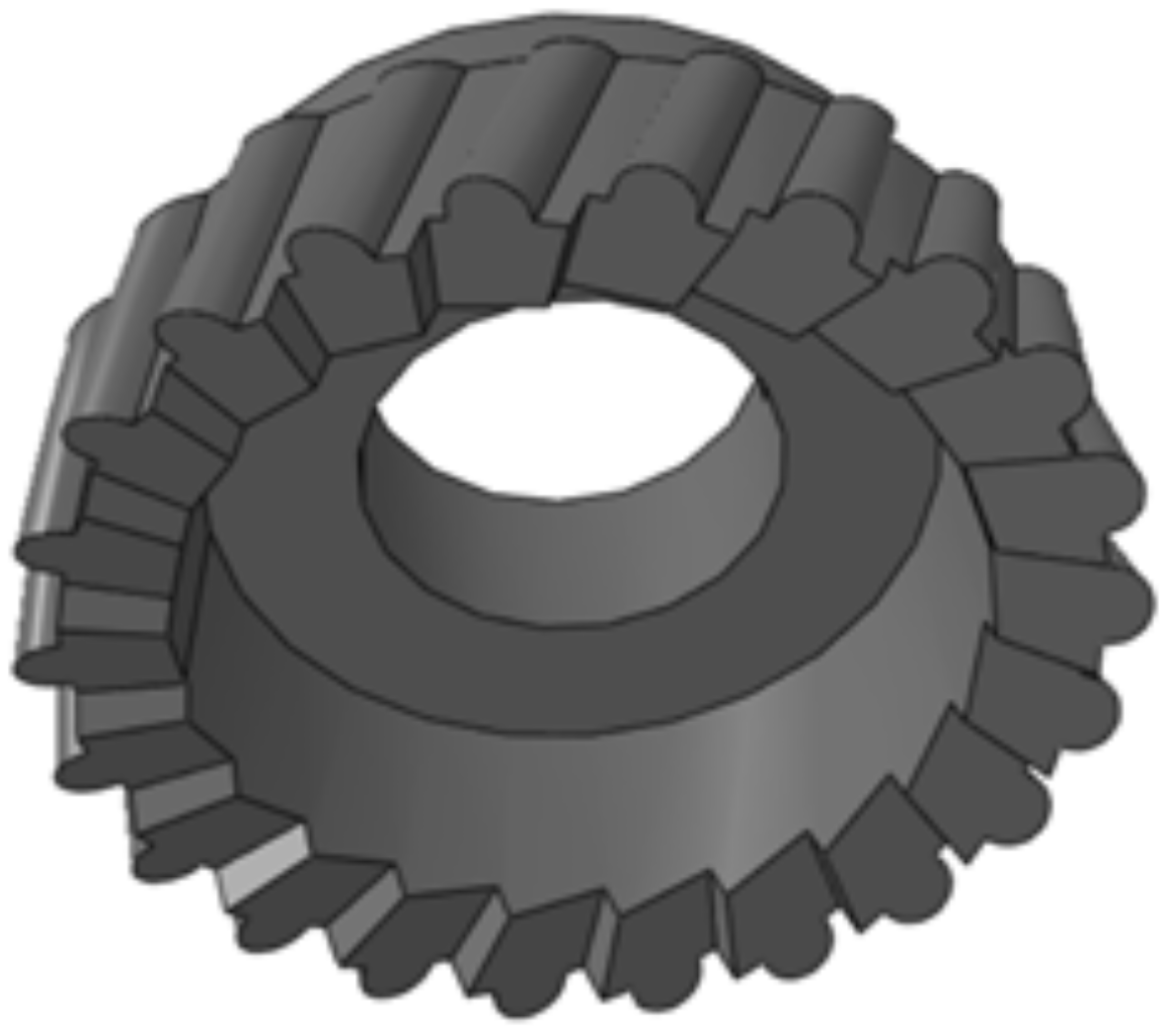
Skiving cutter model.

The rake face is designed as an inclined plane, and the flank face is designed as a cubic B-spline surface which is formed by several error-free cutting edges. The completed 3D model of the cutter is shown in (Fig. 1).

### Simplification of simulation model

**Fig. 2:**
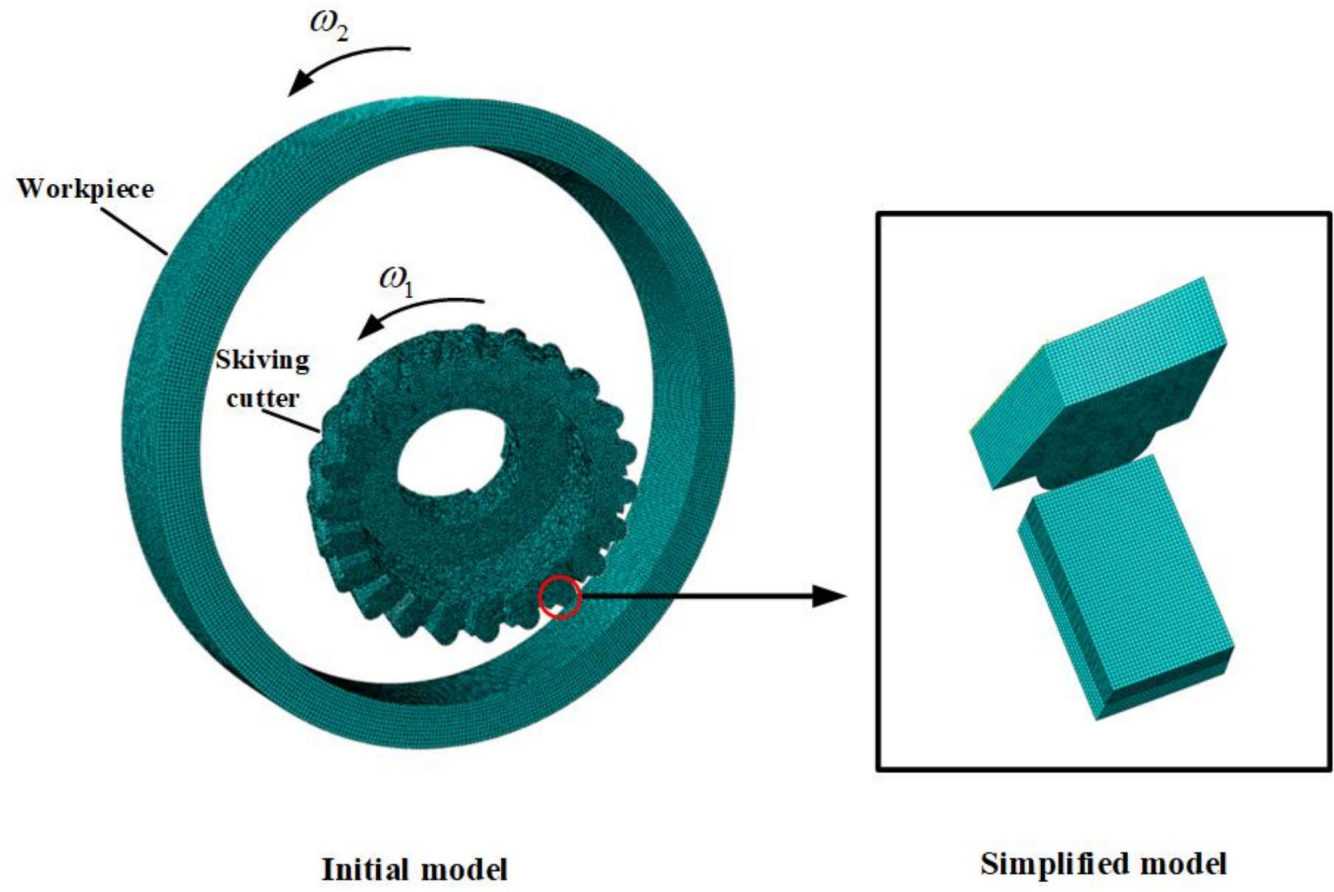
Modeling and simplification of finite element simulation regarding the gear skiving motion.

To save computation time, the simulation model is simplified to calculate the single tooth involved in cutting, considering that the cutting process of each tooth is independent, as shown in (Fig. 2). The advanced hexahedral algorithm is used to control the mesh properties of skiving cutter and workpiece. The mesh element type is C3D8T thermally coupled hexahedral with a geometric order of linear interpolation. In the Boundary Condition Manager of Abaqus, the kinematic loads of the cutter and the workpiece are added respectively to the reference points on the centre axes of the cutter and the workpiece. At the same time, the initial temperature is set as 20 ℃, which is equal to the room temperature, in the Predefined Field Manager.

### Material properties and analysis step

The material of the pin wheel housing is steel 25CrMo4, and the material properties are shown in (Table 3).

**Table 3:** Material properties of steel 25CrMo4.

Density/*kg*/*m*^3^	Elasticity modulus/*MP*_*a*_	Poisson’s ratio	Specific heat/(*J*/(*kg.k*))	Thermal conductivity/(*w*/(*m.k*))
$7850$	$2.1 \times {10^5}$	$0.33$	$450$	$42$

The simulation model adopts Johnson-Cook damage model, whose constitutive equation^13^ of the workpiece material is as follows:

1 $$\bar {\sigma }=\left( {A+B{\varepsilon ^n}} \right)\left( {1+c\ln \frac{{\dot {\varepsilon }}}{{{\varepsilon _0}}}} \right)\left[ {1-{{\left( {\frac{{T-{T_{mo}}}}{{{T_{ml}}-{T_{mo}}}}} \right)}^m}} \right]$$

Where, $\bar {\sigma }$ means equivalent stress. *A* means yield strength of the material. *B* means hardening modulus. *n* means coefficient of strain strengthening. $\varepsilon$ means equivalent plastic strain. *c* means sensitivity coefficient of strain rate. *m* means softening index of temperature. $\dot {\varepsilon }$ means strain. ${\varepsilon _0}$ means reference plastic strain rate. ${T_{{\text{ml}}}}$ means melting temperature of the material. ${T_{mo}}$ means reference temperature. The above constitutive parameters can be obtained from the material library in Deform software, as shown in (Table 4).

**Table 4:** Parameters of the Johnson-Cook constitutive model.

A(*MP*_*a*_)	B(*MP*_*a*_)	*n*	*m*	*c*	${{{T_{ml}}} \mathord{\left/ {\vphantom {{{T_{ml}}} {^{^\circ }c}}} \right. \kern-0pt} {^{^\circ }c}}$	${T_{{\text{mo}}}}$
$714$	$563$	$0.518$	$0.698$	$0.037$	$1600$	$25$

The material of the skiving cutter is tungsten carbide WC, and the material parameters are shown in (Table 5).

**Table 5:** Material properties of the skiving cutter.

Density/*kg*/*m*^3^	Elasticity modulus/*MP*_*a*_	Poisson’s ratio	Specific heat/(*J*/(*kg.k*))	Thermal conductivity/(*w*/(*m.k*))
$15,700$	$6.5 \times {10^5}$	$0.25$	$470$	$59$

The force on the skiving cutter mainly comes from radial direction, tangential direction and axial direction. Then, the whole cutting force is obtained by combining the above three forces.

### Experimental verification of simulation data

**Fig. 3:**
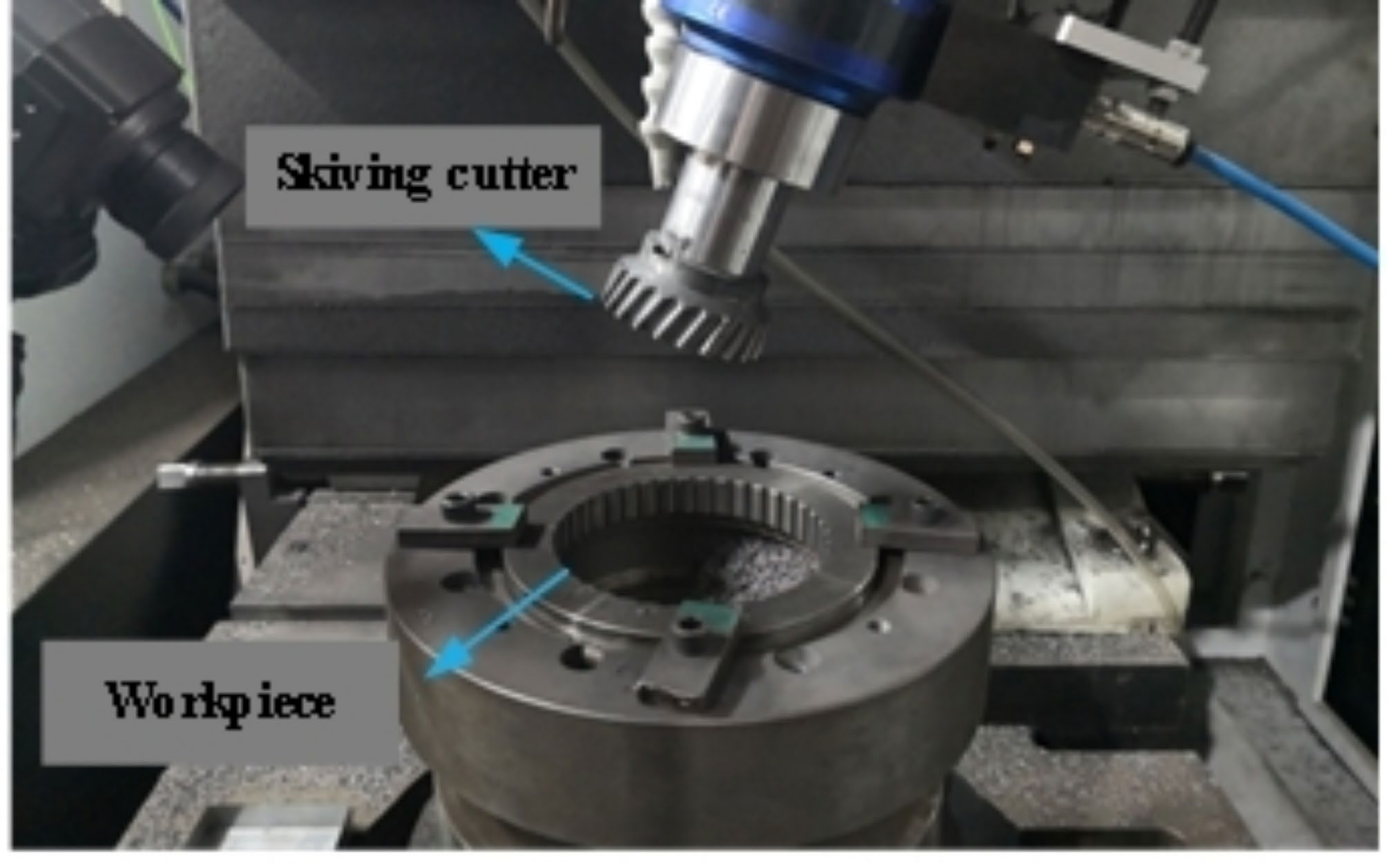
Gear skiving experiment.

To validate the accuracy of the simulation results obtained from the simplified model, a machining experiment of internal circular arc tooth was carried out, as shown in (Fig. 3). During the experiment, the angular speed of the skiving cutter is 73.26 rad/s, while the angular speed of the workpiece is 40.29 rad/s. The feed rate is 0.2 mm/r, and the cutting depth is set as 0.1 mm.

**Fig. 4:**
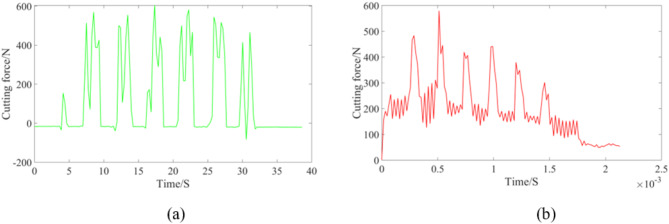
Comparison of experiment and finite element simulation. (**a**) Experiment (**b**) Finite element simulation.

The data of cutting force were collected as shown in (Fig. 4a). The cutting force waveform obtained by finite element simulation under the same process parameters is presented in (Fig. 4b).

By comparing the above data, it can be found that the fluctuation feature is consistent under the same process parameters. The peak cutting forces of the two cases are both approximately equivalent to 600 N. This result proves that the simplified finite element model is accurate and reliable.

## Simulation experiment and result analysis

### Single-factor experiment scheme

In order to explore the variation rules of cutting force and cutting temperature with the above process parameters, a single-factor experiment is carried out by the method of controlling variables. The main process parameters of the experiment include the angular speeds of the skiving cutter and the workpiece, the feed rate of the workpiece, and the cutting depth. Where, the ratio between the angular speed of skiving cutter and the angular speed of workpiece meet the following relationship:

2 $$\frac{{{v_d}}}{{{v_g}}}=\frac{{{n_g}}}{{{n_d}}}$$

Where, ${v_d}$ means the angular speed of skiving cutter. ${v_g}$ means the angular speed of workpiece. ${n_g}$ means the number of internal arc teeth. ${n_d}$ means the number of skiving cutter teeth. The experiment scheme is shown in (Table 6).

**Table 6:** Single-factor experiment scheme.

Number	Angular speed/(rad/s)	Feed rate/(mm/r)	Cutting depth/(mm)
1 ~ 3	95.14,237.84,380.54	0.1	0.6
4 ~ 6	157	0.1,0.6,1.1	0.6
7 ~ 9	95.14	0.1	0.7,0.9,1.1

Due to the rotation ratio between the skiving cutter and the workpiece is constant, only the skiving cutter’s angular speed is taken as an independent variable. The angular speed of the skiving cutter varies within the range from 95.14 to 380.54 rad/s. The feed rate varies within the range from 0.1 to 1.1 mm/r, while the cutting depth varies within the range from 0.7 to 1.1 mm. Three levels are selected for each parameter at equal intervals within the given range.

### Analysis of the influence of process parameters on cutting force

**Fig. 5:**
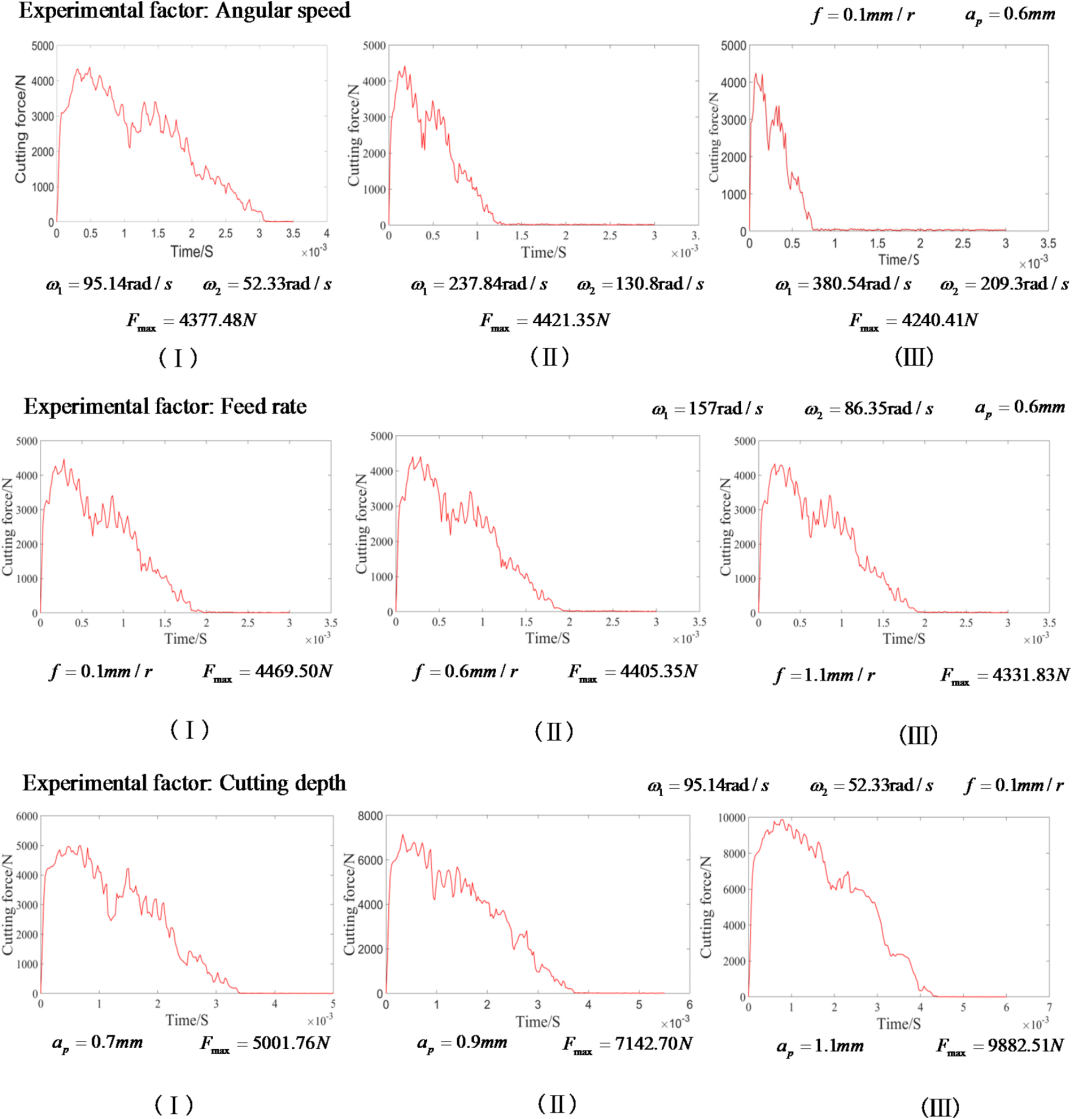
Waveform diagram of cutting force related to single factor.

After verifying the reliability of the simplified finite element simulation model, a single-factor experiment is carried out according to the scheme in Sect. 3.1. The objective is to investigate the impact of individual process parameter on the cutting force and cutting temperature. The simulation results of the cutting force of the skiving cutter are shown in (Fig. 5).

It can be seen from Fig. 5 that the angular speed of the skiving cutter has significant impact on the cutting force waveform. The peak cutting force initially increases from 4377.48 to 4421.35 N and then decreases to 4240.41 N, showing little variation although the cutting time decreases from 0.003 to 0.00075 s.

When the angular speed of the skiving cutter is set as 157 rad/s and the cutting depth is 0.6 mm, the fluctuation time range of the cutting force remains at about 0.002 s. It is shown that the variation of feed rate has minimal impact on the time of single-pass cutting, while the peak cutting force decreases from 4469.50 N to 4331.83 N, showing a trend of gradually decreasing.

Similarly, the impact of the cutting depth on cutting force waveform is analyzed. When the cutting depth changes from 0.7 mm to 1.1 mm, the peak cutting force increases from 5001.76 N to 9882.51 N, and the single-pass cutting time changes slightly from 0.0035 s to 0.0042 s. The cutting depth shows more obvious impact on peak cutting force than the other two process parameters.

### Theoretical analysis of cutting force

To further explore the influence mechanism of cutting force, theoretical calculation of cutting force is conducted under gear skiving motion. The shear area is analyzed according to simulation results, and the cutting rake angle is calculated. Then the cutting force is calculated using the theoretical formula, and the effects of shear area and rake angle on the cutting force are analyzed.

**Fig. 6:**
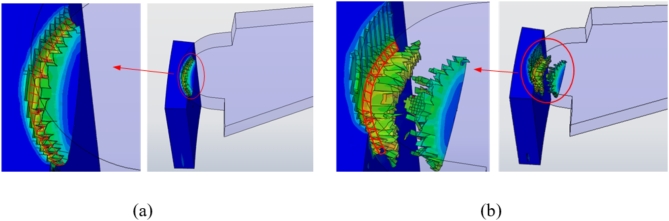
Shear area in gear skiving. (**a**) Shear area${A_o}$ of the first stage (**b**) Shear area ${A_i}$ of the second stage.

**Fig. 7:**
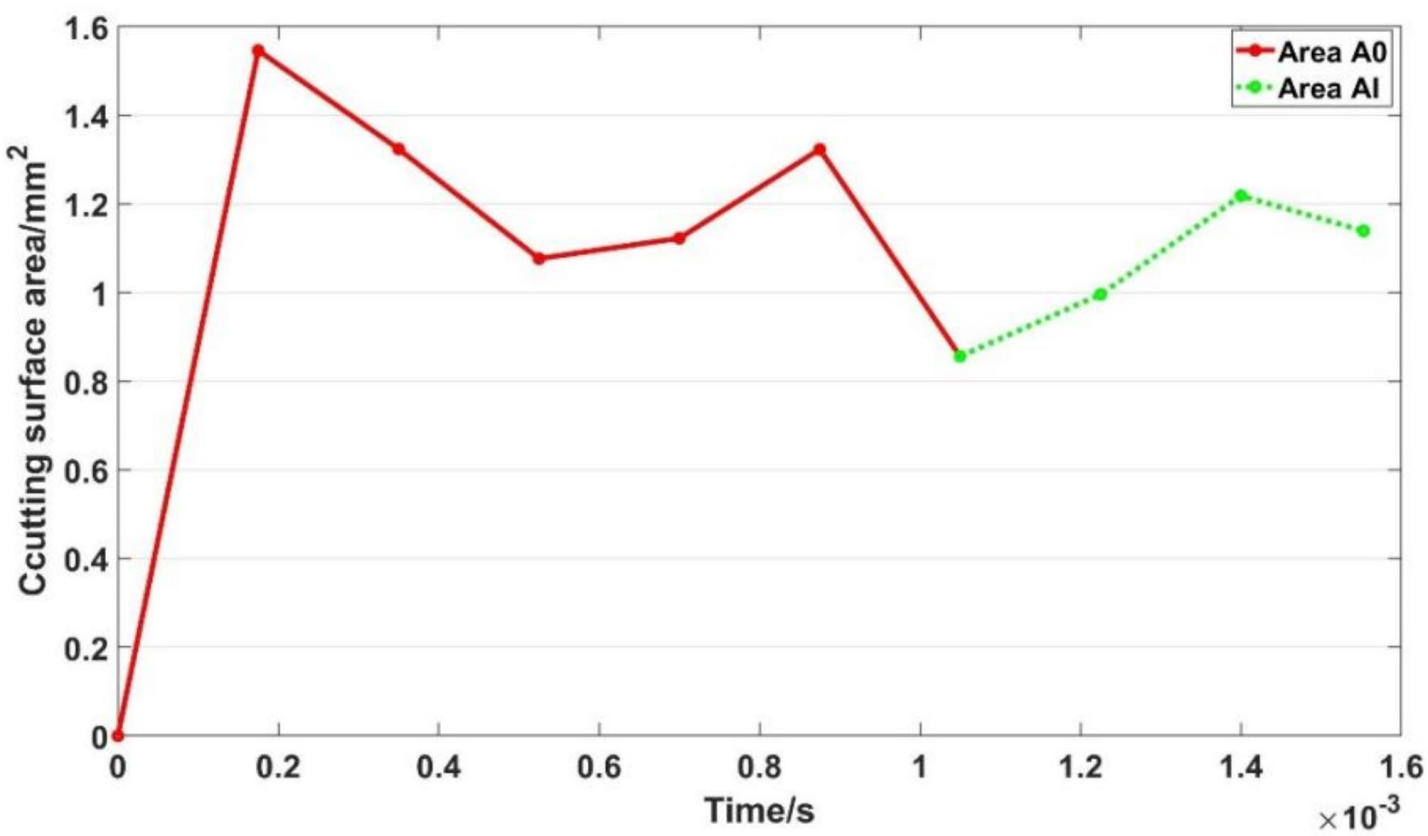
Shear area curve in different stages.

According to the cutting principle of gear skiving, the process of material removal is divided into two stages. The first stage is the shedding of the surface material, and the second stage is the shedding of the internal material. Therefore, the shear area *A* should also be calculated in two parts, specifically the shear area ${A_o}$ when the surface material falls off and the shear area ${A_i}$ when the internal material falls off. The shear area ${A_o}$ is composed of two arc-shaped regions, as shown in the red region in (Fig. 6a). The shear area ${A_i}$ of the second stage is shown in (Fig. 6b). The change curve of shear area is shown in (Fig. 7).

It can be seen that the shear area ${A_o}$ increases firstly and then decreases, and then continues to increase. The maximum area is about 1.546 mm^2^ and the minimum area is about 1.077 mm^2^. The fluctuation range is 0.469 mm^2^, and the chips generated by the falling material are completely detached at the time of 0.00085 s. At about 0.001 s, the workpiece enters the second cutting stage, and the internal material begins to fall off to produce chips. The shear area ${A_i}$ gradually increases firstly and then gradually decreases. The maximum area of ${A_i}$ is about 1.219 mm^2^ and the minimum is about 0.856 mm^2^. The fluctuation range is about 0.363 mm^2^. It is concluded that the first cutting stage has a larger shear area and a greater amount of change.

On the other hand, the cutting rake angle ${\gamma _\text{p}}$ is calculated by referring to Eq. (3)^12^.

3 $${\gamma _\text{p}}=\arccos (\sqrt {\frac{{{{(v_{{12}}^{e}.{n_p})}^2}}}{{{{(v_{{12}}^{e}.{n_p})}^2}+{{({N_1}.{n_p})}^2}}}} )$$

**Fig. 8:**
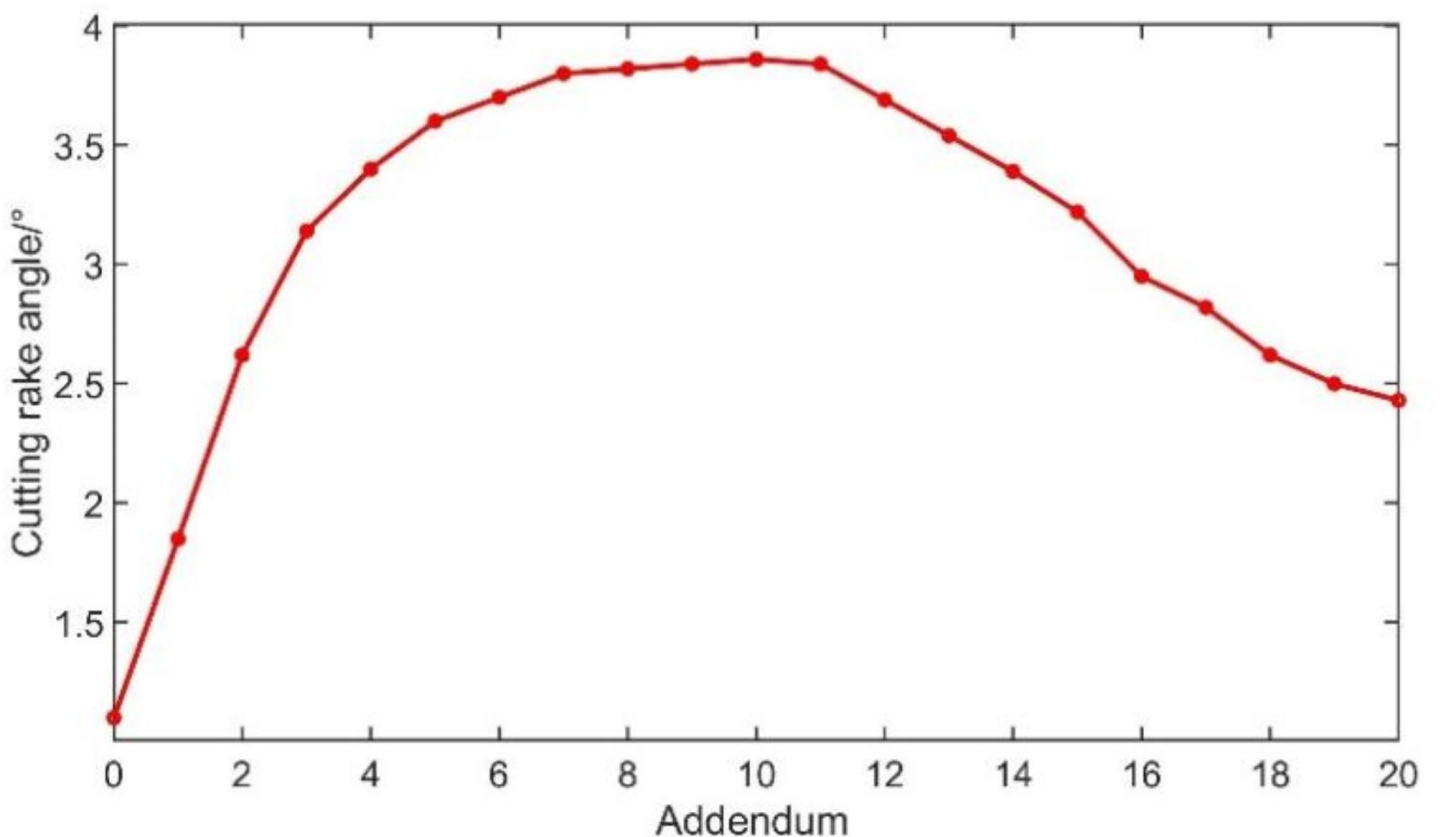
Cutting rake angles at different positions of the cutting edge.

Where, ${\gamma _\text{p}}$ means the cutting rake angle in the workpiece coordinate system, $v_{{12}}^{e}$, ${{\text{n}}_{\text{p}}}$ and ${{\text{N}}_{\text{1}}}$ mean respectively the relative velocity unit vector, the normal vector of the rake face of the skiving cutter, and the vector of the intersection direction of the main cutting edge and the base plane. Finally, the cutting rake angles at 20 points on the cutting edge of the skiving cutter are obtained as shown in (Fig. 8).

It can be seen that the cutting rake angle at the tooth tip is the largest, and the maximum is about 3.86 °. The cutting rake angle decreases gradually from the tooth tip to the both ends of the cutting edge.

Finally, according to the relationship of energy conservation in gear skiving, the cutting force *F* can be calculated by Eq. (4)^5^:

4 $$F={\sigma _s}A.\frac{{\cos {\gamma _e}}}{{\cos ({\phi _e} - {\gamma _e})}}+\frac{{{\sigma _s}.A.\sin {\beta _e}}}{{\cos ({\beta _e}+{\phi _e} - {\gamma _e})}}.\frac{{\sin {\phi _e}}}{{\cos ({\phi _e} - {\gamma _e})}}$$

Where, ${\beta _e}$ means the friction angle, ${\phi _e}$ means the shear angle which can be derived from the cutting rake angle ${\gamma _\text{p}}$, and ${\sigma _s}$ means yield strength of the workpiece.

**Fig. 9:**
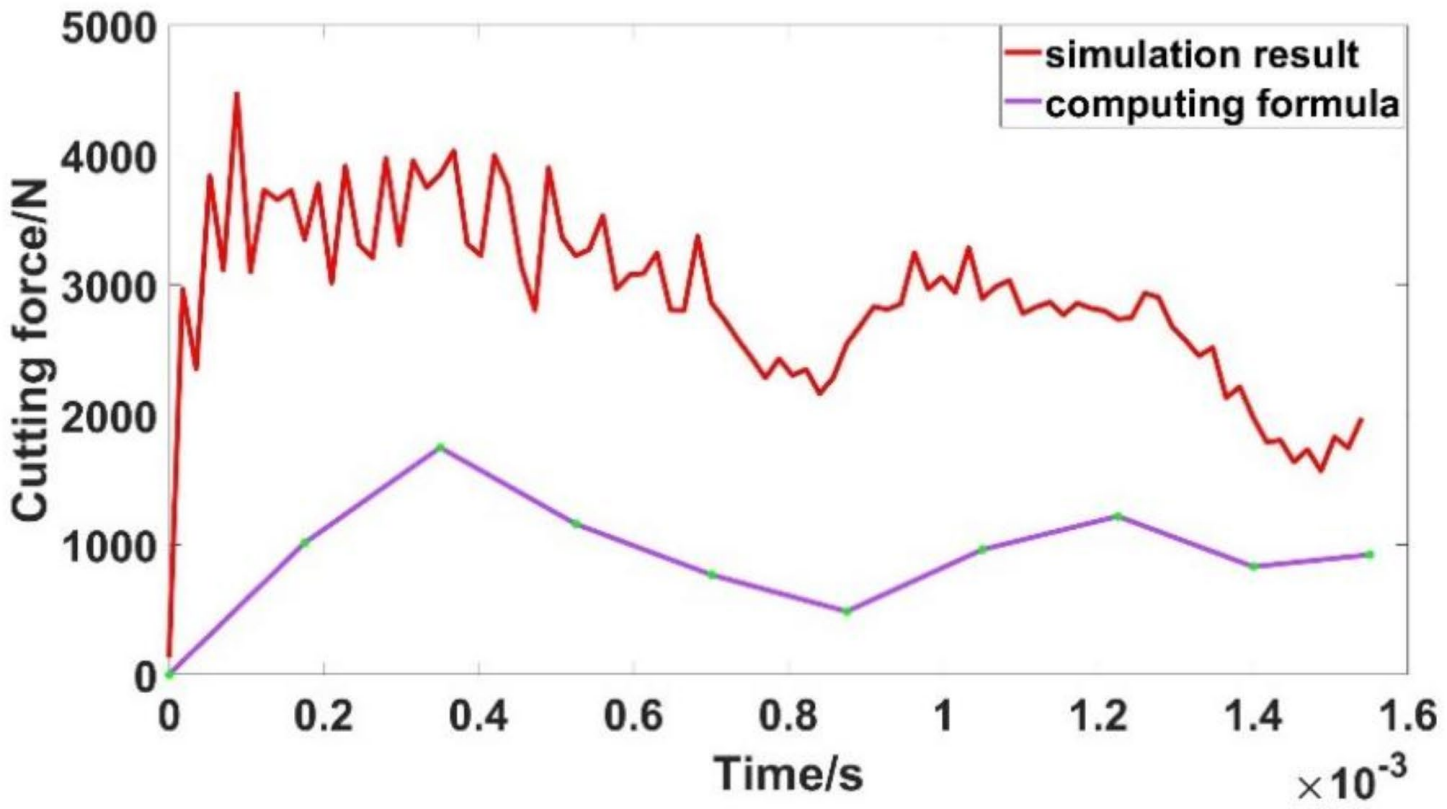
Comparison of cutting force curves.

In the total cutting time, several points are taken at equal intervals to obtain the values of the above variables. The cutting force can be calculated by substituting these values into Eq. (4). The calculated cutting force and the result of finite element simulation are shown in (Fig. 9).

It can be found that the change rule of the calculated cutting force and the result obtained by finite element simulation is basically the same, but the value of the calculated cutting force is smaller than that obtained by finite element simulation. The main reason for the deviation comes from the measurement of the shear area in the simulation model and the adoption of scaling factor. The cutting force waveform obtained by calculation formula shows that the ratio of the shear angle ${\phi _e}$ and the shear area *A* is relatively large when the cutting force is large, and the cutting force is proportional to the ratio of them. At the intermediate stage of the cutting process, the cutting rake angle is relatively large, resulting in smaller cutting force.

In summary, this paper employs finite element simulation, theoretical calculation, and experiment to obtain the cutting forces in gear skiving. It can be observed that the cutting force values obtained through finite element simulation are relatively close to those obtained through experiment, demonstrating that the accuracy of the finite element simulation meets the requirements. The cutting force values obtained from the theoretical model, due to simplification and approximation, deviate to some extent from those obtained from the finite element simulation. However, the theoretical model reveals the underlying mechanism of the generation of skiving cutting forces, and it can demonstrate the direct effect of cutter angles and process parameters on the cutting forces. The above three methods can be jointly used for the analysis of skiving characteristics.

### Analysis of the influence of process parameters on cutting temperature

**Fig. 10:**
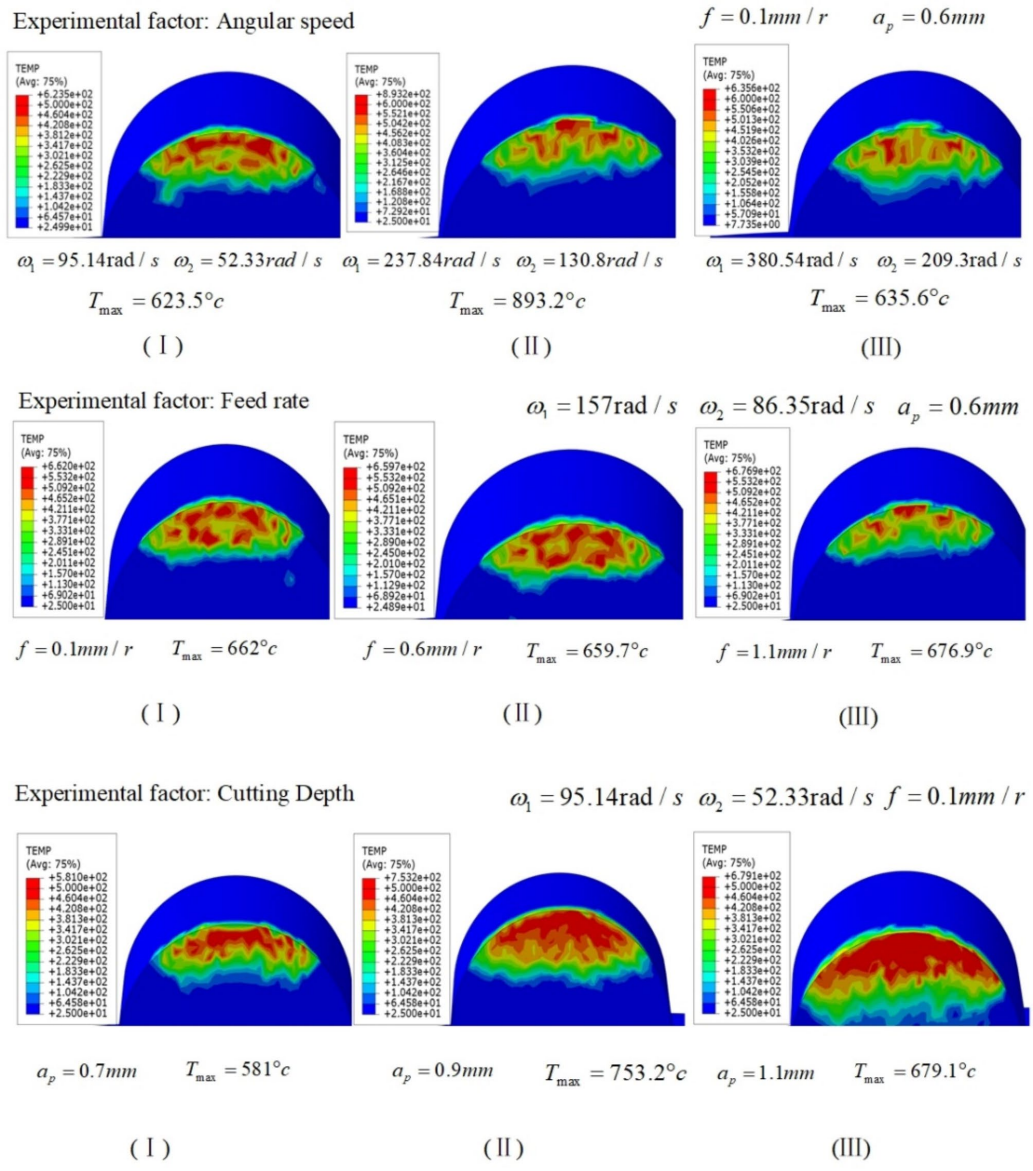
Cutting temperature cloud map.

On the other hand, the cutting temperature is simulated by the finite model, as shown in (Fig. 10).

From the above temperature cloud map, it can be seen that the peak cutting temperature increases from 623.5 to 893.2 ℃, afterwards decreases to 635.6 ℃ when the angular speed of the skiving cutter increases. When the feed rate increases, the peak cutting temperature decreases from 662 to 659.7 ℃, and then increases to 676.9 ℃. When the cutting depth increases, the peak cutting temperature increases from 581 to 753.2 ℃, and subsequently decreases to 679.1 ℃. The angular speed of the skiving cutter has the most significant impact on cutting temperature.

### Analysis of tool wear

**Fig. 11:**
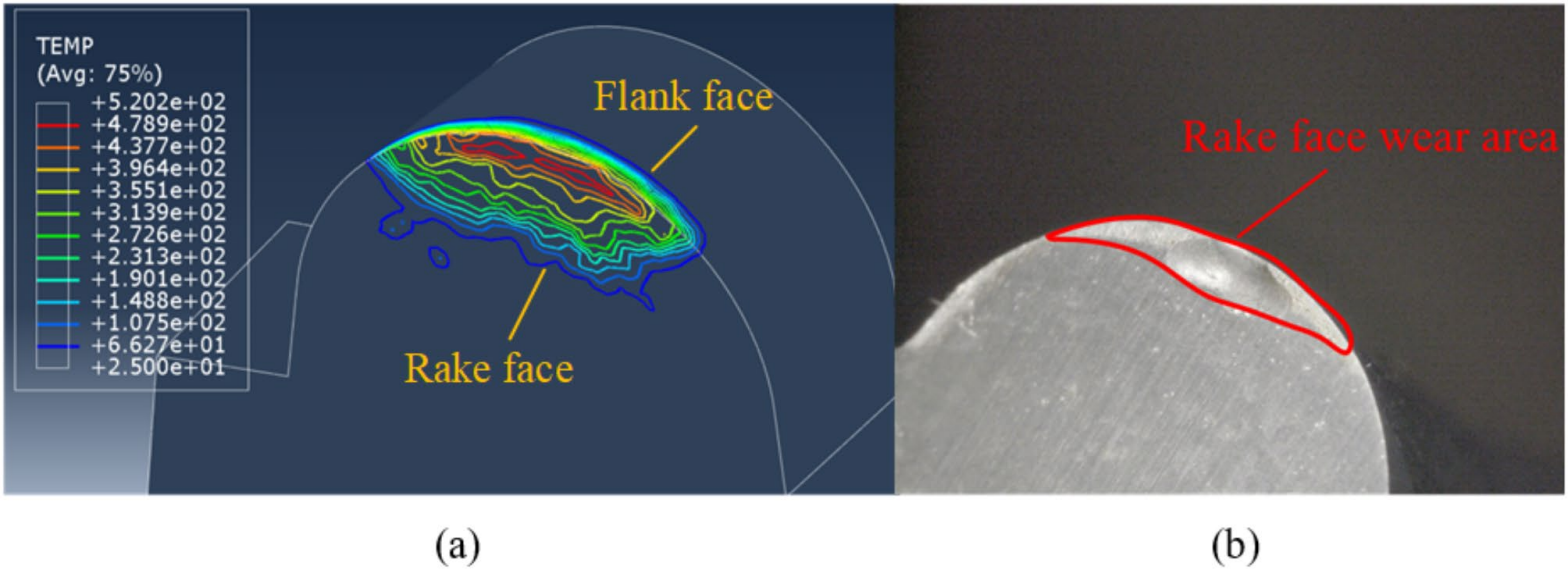
Isotherm cloud map and wear of skiving cutter. (**a**) Isotherm cloud map (**b**) Cutter wear.

To visually illustrate the temperature distribution on the surface of the skiving cutter, an isotherm cloud map is drawn, as shown in (Fig. 11a). The map reveals that the peak temperature is 520.2 ℃. The isotherms near the cutting edge on the flank face are denser, indicating significant temperature dropping.

The isotherms on the rake face are relatively sparse, but the red isotherms predominantly distributed in this region. This indicates that the rake face has a large area of high-temperature zone, which is unevenly distributed near the cutting edge. Therefore, when skiving internal arc teeth, the rake face near the cutting edge is more prone to wear compared with the flank face, and the wear distribution is uneven.

The physical image of the skiving cutter is displayed in (Fig. 11b). It is evident that the wear zone on the rake face basically coincides with the high-temperature zone in the isotherm cloud map. The wear distribution is not uniform, which is consistent with the conclusion obtained from the isotherm cloud map.

### Orthogonal experimental analysis of process parameters

In order to further analyze the influence law of process parameters on cutting force and cutting temperature, a orthogonal experiment^14^ scheme is established, which has 3 factors and 3 levels. The factors are represented by A, B, and C respectively. Three levels are taken for each factor, and different levels under each factor are represented by A_1_, A_2_, A_3_, B_1_, B_2_, B_3_, C_1_, C_2_, C_3_, as shown in (Table 7).

**Table 7:** Factors and levels of orthogonal experiment.

Levels	Factor A (cutter angular speed/(rad/s))	Factor B (feed rate/(mm/r))	Factor C (cutting depth/(mm))
1	95.14	0.1	0.4
2	237.84	0.4	0.6
3	380.54	0.7	0.8

According to the principle of orthogonal table, 9 groups of parameters are selected and the cutting force and the cutting temperature are obtained by the finite element analysis, which are recorded in (Table 8).

**Table 8:** Orthogonal experiment results.

Experiment schemes	Angular speed (rad/s)	Feed rate (mm/r)	Cutting depth (mm)	Mean cutting force (N)	Peak cutting force (N)		Peak cutting temperature (℃)
A_1_B_1_C_1_	95.14	0.1	0.4	1345.62	2800.87		561.1
A_1_B_2_C_2_	95.14	0.4	0.6	2319.17	4365.50		609.7
A_1_B_3_C_3_	95.14	0.7	0.8	3277.97	6317.05		614.7
A_2_B_1_C_2_	237.84	0.1	0.6	2115.22	4421.34		893.2
A_2_B_2_C_3_	237.84	0.4	0.8	3618.93	6742.52		663.1
A_2_B_3_C_1_	237.84	0.7	0.4	1314.27	2745.07		678.5
A_3_B_1_C_3_	380.54	0.1	0.8	3321.07	6816.26		687.0
A_3_B_2_C_1_	380.54	0.4	0.4	1237.24	2721.92		616.4
A_3_B_3_C_2_	380.54	0.7	0.6	1871.03	4295.39		644.0

#### Influence degree of each factor on cutting force

Based on the mean and peak data of cutting force recorded in Table 8, the mean value for each level and range value under each factor are recorded by the method of range analysis^15^, as shown in (Table 9).

**Table 9:** Horizontal mean and range values of cutting force for each factor.

Factors	Mean cutting force/N			Peak cutting force/N		
	Angular speed	Feed rate	Cutting depth	Angular speed	Feed rate	Cutting depth
Mean K_1_	2314.25	2260.64	1299.04	4494.47	4679.49	2755.95
Mean K_2_	2349.47	2391.78	2101.81	4636.31	4609.98	4360.74
Mean K_3_	2143.11	2154.42	3405.99	4611.19	4452.50	6625.28
Range R	206.36	237.36	2106.95	141.84	226.99	3869.32

According to the range value R of each factor in the table, it can be known that the degree of the influence of each factor on the mean cutting force and peak cutting force is as follows: cutting depth > feed rate > angular speed. In addition, the degree of the influence of cutting depth is much greater than that of angular speed and feed rate due to much greater range value R.

In Table 9, K_1_, K_2_ and K_3_ respectively represent the mean value corresponding to each level set in the orthogonal experiment. The mean cutting force shows K_3_ < K_1_ < K_2_ under angular speed. Therefore, the optimal levels of the angular speed of the skiving cutter and the workpiece for mean cutting force are 380.54 rad/s and 209.30 rad/s respectively. By the same method, the optimal level of feed rate is obtained as 0.7 mm/r, and the optimal level of cutting depth is obtained as 0.4 mm.

In the same way, it can be obtained that the optimal levels of the angular speed of the skiving cutter and the workpiece for the peak cutting force are 95.14 rad/s and 52.33 rad/s respectively. The optimal level of the feed rate is 0.7 mm/r, and the optimal level of the cutting depth is 0.4 mm.

#### Influence degree of each factor on cutting temperature

According to the experiment data recorded in Table 8, the method of range analysis is used to analyze the influence of each factor on the peak cutting temperature, and the results are recorded in (Table 10).

**Table 10:** Horizontal mean and range values of cutting temperature for each factor.

Factors	Angular speed (rad/s)	Feed rate (mm/r)	Cutting depth (mm)
Mean K1	595.17	713.77	618.67
Mean K2	744.93	629.73	715.63
Mean K3	649.13	645.73	654.93
Rang R	149.76	84.03	96.97

The values of R corresponding to the three factors show: angular speed > cutting depth > feed rate. This result indicates that the angular speed has the greatest impact on the peak cutting temperature, followed by the cutting depth, and finally the feed rate.

In Table 10, the optimal level of the angular speed of the skiving cutter can be obtained as 95.14 rad/s, and the angular speed of the workpiece is 52.33 rad/s according to K1 < K3 < K2. Similarly, the optimal level of the feed rate is 0.4 mm/r, and the optimal level of the cutting depth is 0.4 mm.

## Cutting force and cutting temperature prediction

In order to further optimize the process parameters, this paper uses BP neural network to build the prediction model of cutting force and cutting temperature.

### Construction of Bp neural network

**Fig. 12:**
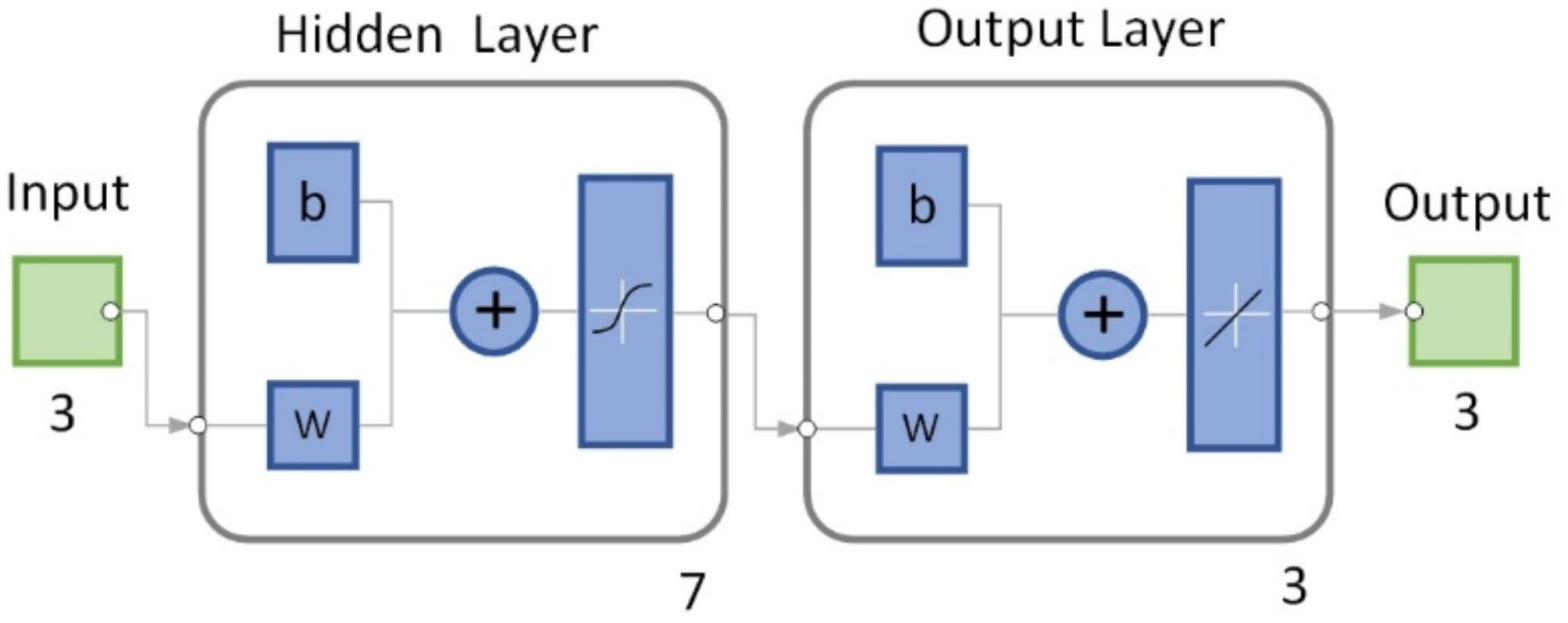
BP neural network structure.

The data obtained from the above orthogonal experiment are used as the training data of the neural network^16^. Because the orthogonal experiment contains 3 parameters and 3 results, the BP neural network should contain 3 input layers and 3 output layers, and the number of hidden layers should be 7, as shown in (Fig. 12). The training data are divided randomly. The transfer function from the input layer to the hidden layer is Tansig Function, and the transfer function from the hidden layer to the output layer is Purelin Function. The maximum number of iterations is set as 1000, the learning rate is 0.01, and the minimum training error is 0.00001.

**Fig. 13:**
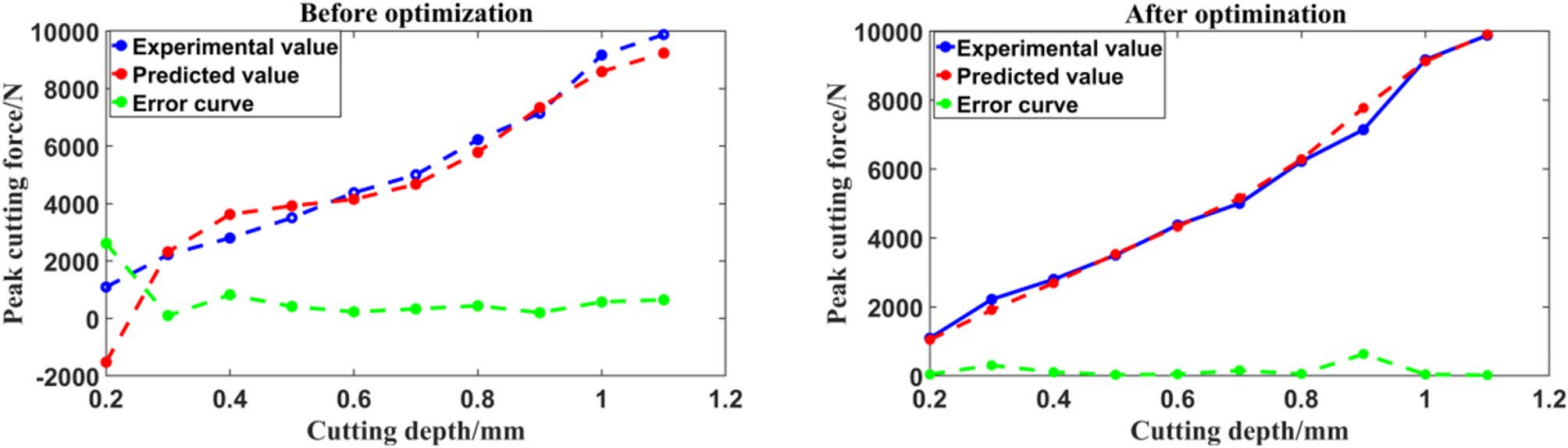
Error comparison before and after optimization by genetic algorithm.

The prediction accuracy of Bp neural network can be improved by optimizing the initial weights and thresholds. In this paper, genetic algorithm^17,18^ is used to optimize the initial weight and threshold of Bp neural network. The prediction accuracy of BP neural network after optimization is compared with that before optimization as shown in (Fig. 13). Where, the experimental curve, the prediction curve and the prediction error of peak cutting force are shown respectively corresponding to different levels of cutting depth.

It can be seen from Fig. 13 that the prediction error curve of the BP neural network optimized by genetic algorithm is smoother, and the fluctuation amplitude is much smaller than that of the BP neural network before optimization, which proves that the genetic algorithm is effective for the optimization of BP neural network.

### Prediction model

In order to eliminate the influence of different data dimension, it is necessary to normalize the input data from orthogonal experiment before BP neural network training. The normalized function is shown in Eq. (5).

5 $${x_{norm}}=2 \times \frac{{(x - {x_{\hbox{min} }})}}{{{x_{\hbox{max} }} - {x_{\hbox{min} }}}} - 1$$

Where, ${x_{norm}}$ means the normalized function value, that is the input data of ${\omega _s}$, ${v_l}$, ${a_p}$ in Eq. (6). *x* means the input data of${\omega _s}$, ${v_l}$, ${a_p}$ before normalization, ${x_{\hbox{min} }}$means the minimum value of ${\omega _s}$, ${v_l}$, ${a_p}$ before normalization, ${x_{\hbox{max} }}$ means the maximum value of ${\omega _s}$, ${v_l}$, ${a_p}$ before normalization.

The normalized data are substituted into the BP neural network optimized by genetic algorithm for training, and the prediction model of the mean cutting force is built as shown in Eq. (6).

6 $$\begin{gathered} {F_a}_{n}=\tan sig(w_{{11}}^{{(2,1)}}{\omega _s}+w_{{12}}^{{(2,1)}}{v_l}+w_{{13}}^{{(2,1)}}{a_p}+b_{1}^{{(2)}}) \times w_{{11}}^{{(3,2)}}+\tan sig(w_{{21}}^{{(2,1)}}{\omega _s}+w_{{22}}^{{(2,1)}}{v_l}+w_{{23}}^{{(2,1)}}{a_p}+b_{2}^{{(2)}}) \times w_{{12}}^{{(3,2)}} \hfill \\ \begin{array}{*{20}{c}} {}&{+\tan sig(w_{{31}}^{{(2,1)}}{\omega _s}+w_{{32}}^{{(2,1)}}{v_l}+w_{{33}}^{{(2,1)}}{a_p}+b_{3}^{{(2)}})} \end{array} \times w_{{13}}^{{(3,2)}}+\tan sig(w_{{41}}^{{(2,1)}}{\omega _s}+w_{{42}}^{{(2,1)}}{v_l}+w_{{43}}^{{(2,1)}}{a_p}+b_{4}^{{(2)}}) \times w_{{14}}^{{(3,2)}} \hfill \\ \begin{array}{*{20}{c}} {}&+ \end{array}\tan sig(w_{{51}}^{{(2,1)}}{\omega _s}+w_{{52}}^{{(2,1)}}{v_l}+w_{{53}}^{{(2,1)}}{a_p}+b_{5}^{{(2)}}) \times w_{{15}}^{{(3,2)}}+\tan sig(w_{{61}}^{{(2,1)}}{\omega _s}+w_{{62}}^{{(2,1)}}{v_l}+w_{{63}}^{{(2,1)}}{a_p}+b_{6}^{{(2)}}) \times w_{{16}}^{{(3,2)}} \hfill \\ \begin{array}{*{20}{c}} {}&+ \end{array}\tan sig(w_{{71}}^{{(2,1)}}{\omega _s}+w_{{72}}^{{(2,1)}}{v_l}+w_{{73}}^{{(2,1)}}{a_p}+b_{7}^{{(2)}}) \times w_{{17}}^{{(3,2)}}+b_{1}^{{(3)}} \hfill \\ \end{gathered}$$

Where, ${F_a}_{n}$ is the mean cutting force predicted before the inverse normalization, $\tan sig$is the transfer function of the neural network, $b_{1}^{{(3)}}$ means the threshold of the output layer, which is shown by Eq. (7). ${\omega _s}$, ${v_l}$, ${a_p}$ are the normalized angular velocity, feed velocity and cutting depth respectively. $w_{{ji}}^{{(2,1)}}$ means the weight from each input layer to the hidden layer, $w_{{kt}}^{{(3,2)}}$ means the weight from each hidden layer to the output layer, and $b_{f}^{{(2)}}$ means the threshold value of each hidden layer, which are shown by Eq. (8) and Eq. (9).

7 $$\tan sig(x) = \frac{2}{{1 + e^{{ - 2x}} }} - 1,b_{1}^{{(3)}} = - 1.1050$$

8 $$w_{{ji}}^{{(2,1)}}=\left[ {\begin{array}{*{20}{c}} {2.0918}&{ - 0.6388}&{1.1822} \\ {0.7700}&{0.4223}&{2.4506} \\ { - 2.1508}&{1.1005}&{ - 1.3876} \\ {2.7413}&{ - 0.8152}&{0.3998} \\ {0.8141}&{ - 1.5683}&{1.9421} \\ {3.3627}&{ - 0.1095}&{0.4403} \\ {0.9397}&{2.2345}&{ - 1.2658} \end{array}} \right],\;b_{f}^{{(2)}}=\left[ {\begin{array}{*{20}{c}} { - 2.9803} \\ { - 1.9509} \\ {~0.6635} \\ { - 0.0082} \\ {1.0905} \\ {1.0102} \\ {2.7667} \end{array}} \right]$$

9 $$w_{{kt}}^{{(3,2)}}=[\begin{array}{*{20}{c}} {0.8853}&{0.1416}&{0.2096}&{ - 1.3396}&{1.1808}&{1.1660}&{1.3735} \end{array}]$$

The mean of ${F_a}$ can be obtained after the inverse normalization of ${F_a}_{n}$ and the inverse normalization function is:

10 $${F_a}=\frac{{({F_{an}}+1) \times ({F_{\hbox{max} }} - {F_{\hbox{min} }})}}{2} - {F_{\hbox{min} }}$$

Where, ${F_{\hbox{max} }}$ and ${F_{\hbox{min} }}$ are respectively the maximum and minimum mean cutting force in the training data.

By the same method, the prediction models of peak cutting force ${F_m}$ and peak cutting temperature ${T_m}$ can be obtained, in which the weights from the hidden layer to the output layer are written as$w_{{qp}}^{{(3,2)}}$and $w_{{gh}}^{{(3,2)}}$ respectively, and the thresholds of the output layer are written as $b_{2}^{{(3)}}$and $b_{3}^{{(3)}}$ respectively. These data are given below.

11 $$\begin{gathered} w_{{qp}}^{{(3,2)}}=[\begin{array}{*{20}{c}} {0.6808}&{0.3308}&{ - 0.2155}&{ - 0.9590}&{1.2836}&{0.5047}&{0.9926} \end{array}]\begin{array}{*{20}{c}} {\begin{array}{*{20}{c}} {}&{} \end{array}}&{} \end{array} \hfill \\ w_{{gh}}^{{(3,2)}}=[\begin{array}{*{20}{c}} { - 0.0206}&{ - 0.7964}&{0.2909}&{ - 0.2334}&{1.3665}&{0.6300}&{ - 0.8153} \end{array}] \hfill \\ \begin{array}{*{20}{c}} {\begin{array}{*{20}{c}} {\begin{array}{*{20}{c}} {\begin{array}{*{20}{c}} {}&{} \end{array}}&{} \end{array}}&{} \end{array}}&{}&{}&{\begin{array}{*{20}{c}} {\begin{array}{*{20}{c}} {}&{} \end{array}}&{} \end{array}} \end{array}b_{2}^{{(3)}}= - 0.7038\begin{array}{*{20}{c}} ,&{b_{3}^{{(3)}}= - 1.0119} \end{array} \hfill \\ \end{gathered}$$

The groups 3, 5 and 7 in the above orthogonal experiment are used to verify the accuracy of the prediction model, and the relative errors are calculated as shown in (Table 11).

**Table 11:** Relative errors of prediction model (%).

Experiment number	3	5	7
Mean cutting force error	8.24	1.41	1.13
Peak cutting force error	3.76	7.44	8.10
Peak cutting temperature error	14.86	1.38	0.67

The errors of the prediction models range from 0.67 to 14.86%, which are lower than 15%, indicating that the prediction models of cutting force and cutting temperature established above have sufficient reliability.

## Multi-objective optimization model

In order to extend the service life of the cutter, the mean cutting force, the peak cutting force and the peak cutting temperature should be controlled at a low level. According to the variation coefficient method^19^ and the data of orthogonal experiment, the weights of the optimization targets are obtained as shown in (Table 12).

**Table 12:** Weights of each optimization target.

Index	Mean cutting force	Peak cutting force	Peak cutting temperature
Mean number	2384.363	4580.658	663.078
Standard deviation	2381.69	4094.34	332.1
Variable coefficient	0.999	0.894	0.501
Weight	0.417	0.373	0.209

Using the weights of the above objectives, a multi-objective optimization model is built in Eq. (12).

12 $$\begin{gathered} \omega _{s} = 95.14\sim 380.54rad/s \hfill \\ v_{l} = 0.1\sim 0.7mm/r \hfill \\ a_{p} = 0.4\sim 0.8mm \hfill \\ FT(\omega _{s} ,v_{l} ,a_{p} ) = \min (0.417 \times F_{a} + 0.373 \times F_{m} + 0.209 \times T_{m} ) \hfill \\ \end{gathered}$$

The optimization model is solved by genetic algorithm, whose parameters are shown in (Table 13).

**Table 13:** Parameters of genetic algorithm.

Population size	Evolutionary algebra	Chromosome length	Crossover probability	Mutation probability
80	100	20	0.7	0.01

**Fig. 14:**
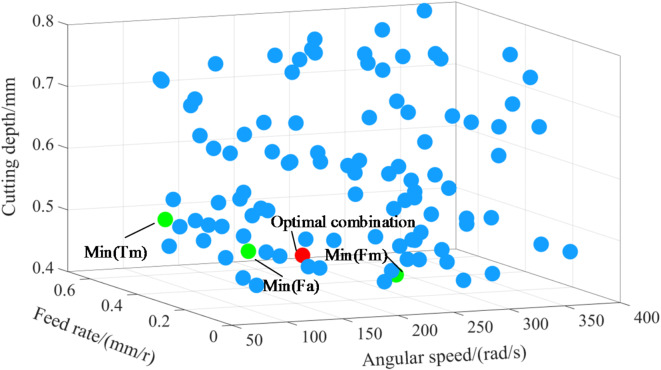
Multi-objective optimization by genetic algorithm.

The evolutionary point diagram of the multi-objective optimization is shown in (Fig. 14). The blue points in the diagram represent 80 individuals in the initial population, which are the random combinations of process parameters to be optimized. The green points represent the optimal values of each single-objective before evolution, that is, the optimal parameter combinations for mean cutting force, peak cutting force and peak cutting temperature respectively as marked in the figure. The red point is the optimal parameter combination after multi-objective optimization by genetic algorithm, where the mean cutting force, the peak cutting force and the peak cutting temperature are taken as optimization targets at the same time.

**Fig. 15:**
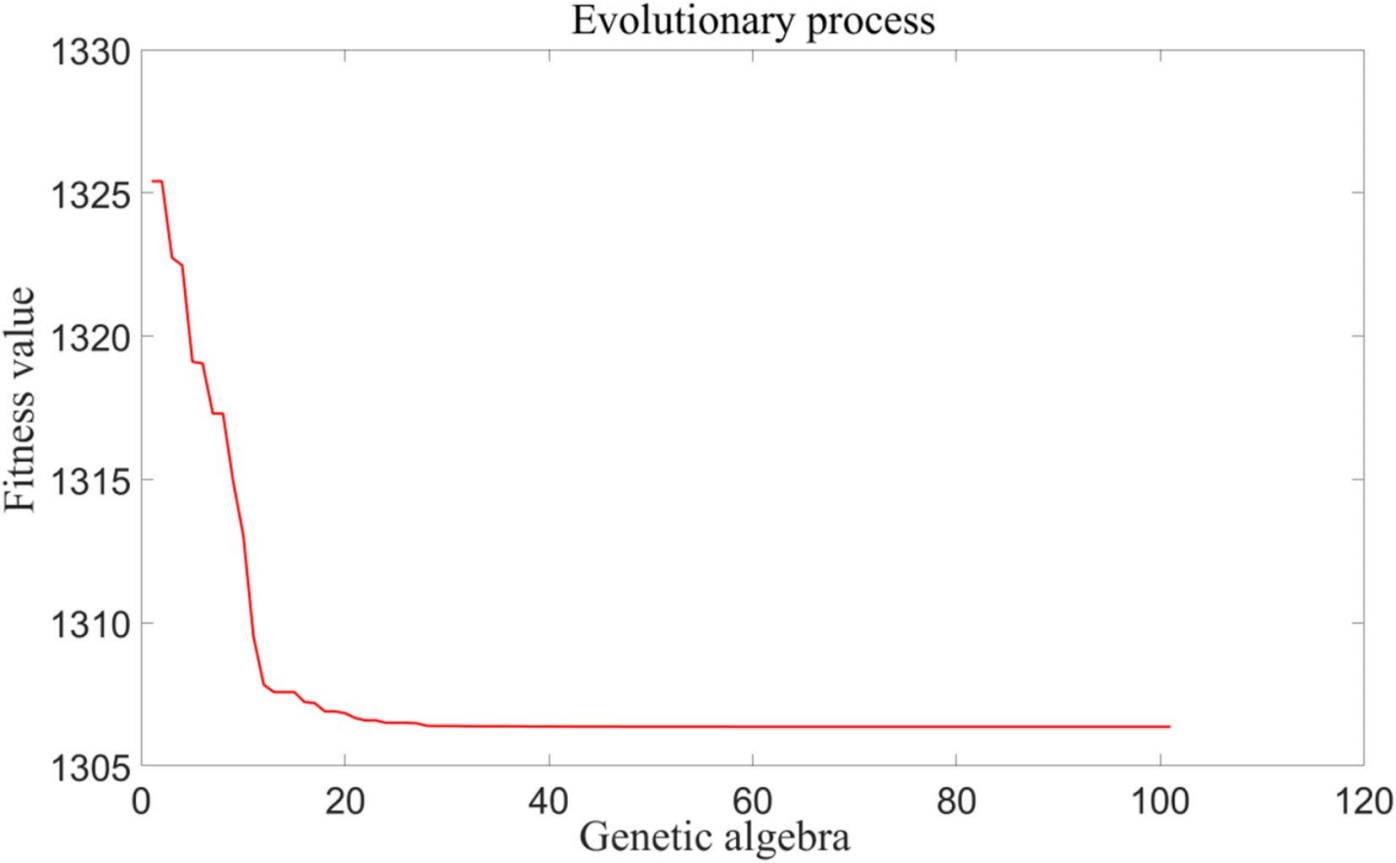
Fitness evolution process.

The evolutionary iterative process is shown in Fig. 15, and the minimum fitness value is 1306.38. The optimal parameter combination is then obtained as follows: angular speed 223.082 rad/s, feed rate 0.513 mm/r, cutting depth 0.431 mm. These parameters are substituted into the prediction model in Sect. 4.2, and the mean cutting force is obtained as 962.749 N, the peak cutting force is 2155.726 N, and the peak cutting temperature is 482.377 ℃. Compared with the results of orthogonal experiment, the values of optimization targets are smaller, the mean cutting force is reduced by 274.5 N, the peak cutting force is reduced by 566.2 N, and the peak cutting temperature is reduced by 78.8 °C. These results indicate that the proposed optimization method for skiving parameters is effective.

## Conclusions

A finite element model of gear skiving for internal circular arc tooth was established in Abaqus to calculate cutting force and temperature. The influence of angular speed, feed rate and cutting depth on cutting force and temperature was analyzed by single-factor method and orthogonal experiment. Then, the prediction models of cutting force and cutting temperature were built with the help of the neural network optimized by genetic algorithm. At last, a optimal combination of process parameters was obtained by multi-objective optimization. The following conclusions are drawn from the above research.

1. The method of range analysis was used to analyze the results of orthogonal experiments, and it was found that the influence degree of angular speed, feed rate and cutting depth on the mean and peak value of cutting force is: cutting depth > feed rate > angular speed. The influence of cutting depth on cutting force is greater than that of feed rate and angular velocity, so in order to reduce the breaking and rolling of skiving cutter, the cutting depth should be controlled preferentially.
2. The high-temperature areas are concentrated on the rake face near the cutting edge, which reveals the reason why this area is more prone to wear.
3. The influence degree of angular speed, feed rate and cutting depth on the peak cutting temperature is: angular speed > cutting depth > feed rate. Because of significant influence on peak cutting temperature, the angular speed of skiving cutter should be controlled within a reasonable range in order to reduce cutter wear.
4. The optimal parameter combination in the given range is obtained by multi-objective optimization. Under these parameters, the cutting force and the cutting temperature can achieve relatively low values at the same time. This research may provide guidance for reducing cutter wear and fracture.

## Data Availability

The authors confirm that the data supporting the findings of this study are available within the article.
